# Adherence and characteristics of participants enrolled in a standardised programme of patient education and exercises for low back pain, GLA:D® Back – a prospective observational study

**DOI:** 10.1186/s12891-021-04329-y

**Published:** 2021-05-22

**Authors:** Inge Ris, Daniel Broholm, Jan Hartvigsen, Tonny Elmose Andersen, Alice Kongsted

**Affiliations:** 1 Department of Sports Science and Clinical Biomechanics, University of Southern Denmark, Campusvej 55, 5230 Odense M, Denmark; 2 Department of Anaesthesiology, Multidisciplinary Pain Centre, Vejle and Middelfart Hospitals, Østre Hougvej 55, 5500 Middelfart, Denmark; 3 Chiropractic Knowledge Hub, Campusvej 55, 5230 Odense M, Denmark; 4 Department of Psychology, University of Southern Denmark, Campusvej 55, 5230 Odense M, Denmark

**Keywords:** Attendance, Adherence, Low back pain, Fidelity

## Abstract

**Background:**

Low back pain is often long-lasting, and implementation of low-cost interventions to improve care and minimise its burden is needed. GLA:D® Back is an evidence-based programme consisting of patient education and supervised exercises for people with low back pain, which was implemented nationwide in primary care clinics in Denmark. To assess how the intervention was received and factors influencing adherence to the program, we aimed to evaluate participants’ adherence to the intervention and identified characteristics related to the completion of GLA:D® Back. Specifically, we investigated: 1) level of attendance of participants enrolled in the programme, and 2) participant-related factors associated with low attendance.

**Methods:**

Primary care clinicians delivered GLA:D® Back, a standardised 10-week programme of 2 educational and 16 supervised exercise sessions, to patients with low back pain. Attendance was defined as low, medium or high based on self-reported number of attended sessions. Additional participant-reported data included demographic characteristics, pain, prognostic risk profiles, self-efficacy, illness-beliefs, function and clinician-reported physical performance tests. Results for high, medium, low, and unknown attendance were reported descriptively. Odds ratios for low attendance compared to medium/high attendance were calculated by including all baseline factors in a mixed-model logistic regression model.

**Results:**

Of 1730 participants, 52% had high, 23% medium, and 25% low levels of attendance. Level of attendance was not strongly associated with participants’ individual factors, but in combination, prediction of low attendance was fair (AUC 0.77; 95% CI 0.74–0.79). The strongest indicator of low attendance was not completing the baseline questionnaire.

**Conclusions:**

Most participants of a 10-week low back pain programme attended almost all session. Non-response to the baseline questionnaire was strongly associated with low attendance, whereas individual patient characteristics were weakly related to attendance. Not completing baseline questionnaires might be an early indicator of poor adherence in programs for people with persistent low back pain.

**Trial registration:**

The Health Research Ethics for Southern Denmark decided there was no need for ethical approval (S-20172000-93). The Danish data collection has obtained authorisation from the Danish Data Protection Agency as part of the University of Southern Denmark’s institutional authorisation (DPA no. 2015-57-0008 SDU no. 17/30591). The trial was registred at ClinicalTrials.gov NCT03570463.

**Supplementary Information:**

The online version contains supplementary material available at 10.1186/s12891-021-04329-y.

## Background

Low back pain (LBP) is one of the leading causes of years lived with disability worldwide and will affect most people at some point in their life [1, 2]. The personal and societal burden of back pain has increased over the last decades [3]. For many, LBP can best be described as a long-lasting condition with episodic or persistent symptoms [4]. Recently, a call for action was made by international researchers advocating for implementation research aimed at implementing low-cost, evidence-based treatments to improve care and minimise the burden of back pain [5]. In response, a standardised, evidence-based programme was implemented in Denmark in 2018, consisting of group-based patient education and supervised exercises at primary care physiotherapy and chiropractic clinics to improve self-management for people with recurrent or persistent LBP, GLA:D® Back [6, 7]. To monitor implementation, adherence was evaluated, defined as participants’ extent to which they act according to the recommendations of the clinician [8], expressed by the level of attendance to sessions in the intervention. Prior studies have reported dropout rates of programs for various musculoskeletal disorders ranging between 7 and 57% [9]. For LBP treatment specifically, dropout rates appear to vary across interventions with approximately 17% (range 0–43%) in motor control exercise trials [10] to approximately 12% (range 0–27%) in cognitive behavioural treatments [11], while dropout from exercise interventions for chronic pain, in general, is reported to be around 18% [12]. Multiple barriers to attendance in physiotherapy have been identified, including low levels of physical activity, low self-efficacy, depression, anxiety, poor social support and pain during exercise [13]. Specifically, for people with non-specific chronic spinal pain, educational level and kinesiophobia are also related to their level of attendance [14]. Thus, more specific knowledge on predictors of clinic-based attendance to appointments in people seeking care for LBP is needed to improve adherence [15]. The GLA:D® Back programme offers a unique opportunity to study adherence to a standardised programme for LBP patients because all patients enrolled are followed in a clinical registry and fill out questionnaires as part of their participation in GLA:D Back. These findings can help understand the drop out behaviour of LBP patients who seek treatment in primary care and possible needs for improvements and adjustments to increase attendance.

### Aims

This study aimed to investigate: 1) adherence to a 10-weeks programme of patient education integrated with exercises, defined by the number of sessions attended, and 2) patient-related factors associated with low attendance.

## Methods

### Design

This study was conducted as a prospective observational study based on information in the GLA:D® Back register [7]. The sample size was the number of participants enrolled in the GLA:D® Back programme from April 1st, 2018 until February 1st, 2020. The study is part of a comprehensive research programme, studying effects and mechanisms of implementing structured care for LBP [7].

### Setting

The theoretical framework, content, access to the material (additional files), and scientific evidence of the programme is presented in detail elsewhere [6, 7]. In short, the programme was taught to primary care physiotherapists and chiropractors in Denmark during a two-day self-paid course. After attending the course, clinicians were certified to deliver the programme to patients with persistent or recurrent LBP in their clinics. The programme consists of an individual session with clinical testing and goal setting followed by two one-hour group sessions of patient education with a focus on knowledge of back pain, pain behaviour and beliefs, and fear of movement. Next are 8 weeks of bi-weekly one-hour supervised exercise group sessions where key messages (e.g. hurt doesn’t harm, stay active, free movements reduce pain) from the patient education sessions are repeated. Exercise sessions contain a short warmup and eight types of exercises with four levels of difficulty targeting the back, abdominal, buttock and leg muscles as well as exercises for flexibility. Finally, there is a post-treatment evaluation with re-testing and evaluating the goals identified at the onset of the program. Participation requires out-of-pocket expenses for the participants with no uniform prices across clinics [7].

### Participants

Criteria to participate were ≥ 18 years old, having persistent or recurrent LBP with a perceived need for improved self-management (as judged by the clinicians), and consent to be included in the register. Participation was based on shared decision principles [16]. When the clinicians considered a patient eligible to participate, information on GLA:D Back programme content and practicalities including time, costs and duration were communicated to the patient. Based on this information, the patient would decide whether to participate in the programme or not.

### Data collection

Data on participant characteristics and demographics were self-reported pre-treatment. Data about attendance were collected as self-reported participation in the educational and exercise sessions at the three-month follow-up. Data were collected using questionnaires sent automatically via e-mail 3 months after completing the baseline questionnaire which was shortly after completing the programme. Clinicians entered data about their perception of sufficient completion of a participant at the post-treatment session if participants did not attend the post-treatment evaluation session. All data were collected electronically via the Research Electronic Data Capture (REDCap), licensed by the Odense Patient data Explorative Network (OPEN).

### Variables

Attendance to education and exercise sessions was participant self-reported in the 3-months questionnaire as 0, 1, 2, or 3+ patient education sessions and 0, ‘1–5’, ‘6–10’, ‘11–15’, ‘16 or more’ exercise classes. The options of more than 2 patient education sessions and ‘16 or more’ exercise sessions were included in the case of modifications to the programme (standardised as two educational and 16 exercises sessions) or if the participant had attended other interventions perceived as being part of the programme.

Participant characteristics were defined by self-reported information of sex, age, education (none, vocational, higher education < 5 years, higher education ≥5 years), work-situation (ordinary work, unemployed, rehabilitation/flex job, disability pension, retired, student/trainee/other), number of working hours (part-time < 37 h, full-time 37 h, > 37 h), current sick leave, duration of last low back pain episode, co-morbid pain in other areas during the last 2 weeks, any of 15 chronic co-morbid diseases (categorised in ‘none’, ‘1–3’ and ‘> 4’) [17].

Additional questionnaires (see also additional material) and physical tests were used to assess a variety of potential influencing factors. These included

- Intensity of low back and/or leg pain measured on an 11-point scale Numeric Rating Scale (higher scores indicate more pain) [18]
- Risk of poor prognosis measured via the Start Back Screening Tool (low, medium and high risk) a 9-item tool with binary scores 0 or 1. Patients scoring 4 or more are classified as a high risk [19]
- Self-efficacy assessed by the Arthritis Self-Efficacy Scale (ASES) subscales “pain” and “other” translated to Danish (higher scores indicate a higher level of self-efficacy) [20]. ASES contains five items on self-efficacy related to the effect of pain and six items on other symptoms and the ability to control fatigue, being active, mood, daily activities, symptoms, and frustration. Each item is scored on an 0–10 scale (0 = very uncertain; 10 = very certain). ASES was developed for arthritis and fibromyalgia, but not for back pain [20, 21]. For our purpose “arthritis” was changed to “back pain”.
- Patients illness beliefs were measured by the Brief Illness Perception Questionnaire (B-IPQ) [22, 23]. B-IPQ consists of nine items covering eight constructs in numeric scales: consequences, timeline (expectations of prognosis), personal control, treatment control, identity (extent of symptoms), coherence (understanding of symptoms), emotional representation, concerns, and one reported in text: cause. The eight numeric items were summed into one score ranging 0–80 (higher scores indicate more threatening beliefs);
- Back-related disability was measured by the Oswestry Disability Index (ODI) [24, 25]. ODI consists of 10 items scored each on an 0–5 scale with a total score of 0–50 (0 = no disability).
- Physical performance tests of back and abdominal muscles endurance were assessed using the iso-extensor endurance test [26, 27] and trunk flexor endurance test [28, 29] which measure the patients’ ability to maintain a static position up till 3 and 2 minutes, respectively. The instruction of these tests was trained at the 2-day course for clinicians to minimise bias.

The completion of patient-reported questionnaires was defined as “no response”, “partly completed” (did not continue to the final page), “fully completed” (all pages filled out, potentially with some missing values).

In case patients did not attend the post-treatment evaluation session, the clinician evaluated the patients as having received the programme or not, based upon their perception: ‘the patient *completed* the programme, but for some reason, the final tests were not performed’ or ‘the patient *did not complete* the programme, and the final tests were not performed’.

### Definition of high, medium and low attendance

Five members of the GLA:D® Back Development Group indicated via an email-survey what they regarded as a minimum of attendance on the patient education and exercise sessions to have completed the GLA:D® Back intervention. At a consensus meeting between authors (AK, IR, DB), participant-reported attendance at two patient education and a minimum of 10 exercise sessions was defined as completion of the GLA:D Back intervention (Table 1). For those not responding at 3-months follow-up, the information registered by clinicians stating the clinicians’ judgement on whether the intervention had been completed (completed/did not complete) was used and graded as “high attendance” when clinicians stated that the intervention had been completed, while “did not complete” was coded as “low attendance”. Attendance was categorised as “unknown attendance” for participants who did not provide data in the 3-months questionnaire on the number of sessions they had participated in and for whom there was no clinician-recorded attendance (either because of missing values or because the participant did meet for the evaluation session).

**Table 1:**
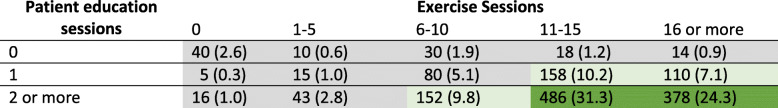
Number of sessions attended, n (%)

Grey = Low attendance; Light Green = Medium attendance; Dark green = High attendance

Numbers relate to *n* = 1555 whose attendance was based on patient-reported number of sessions. For the remainder 175, the level of attendance was clinician-reported and did not include information on numbers of sessions

### Analysis

The level of attendance was described as proportions (%) of participants with known attendance. To evaluate how the consensus derived definitions of levels of attendance matched with clinicians’ judgement, data from participants who responded to the 3-months questionnaire were compared with the clinicians’ registration if the intervention had been completed or not. Proportions in each attendance group (low, medium, high) were calculated within groups of clinicians’ judgements of completion.

To describe participants’ characteristics related to attendance, first, characteristics of low, medium and high attending patients and of those with unknown attendance were described as proportions for categorical measures and medians with interquartile ranges (IQR) for numeric variables. There were no pre-specified hypotheses about associations between attendance and participant characteristics, and therefore the statistical significance of group differences was not tested.

Next, to identify factors associated with attendance independently of other measured factors, we estimated odds ratios (OR) with 95% confidence intervals (CI) of low attendance as compared to medium/high attendance by including all the investigated baseline factors in a mixed model logistic regression model with random effect of clinic. For this analysis, missing values on explanatory variables were assumed to be missing at random and imputed using chained multiple imputations with augmented regression (stata option ‘augment’) to handle the perfect prediction of categorical variables. Because job type and working hours were strongly related, these factors were combined (Ordinary work < 37 h, Ordinary work full-time, Ordinary work > 37 h, Rehabilitation/Flex job < 37 h, Student/Trainee/Other). Further, numeric factors were categorised because of non-linear relationships with the outcome. Model I included all investigated patient characteristics with the lowest level of independent variables as a reference category. Model II collapsed levels with similar ORs within the categorical variables and removed factors with OR close to 1 to obtain a clearer interpretation of the result. For the final analyses, the study population was extended to include patients who did not respond to the baseline questionnaire, and we estimated ORs for low attendance based only on completion of the baseline questionnaire (no response, partly completed, fully completed). The models’ abilities to discriminate between low and medium/high attendance were quantified by Area Under the Receiver Operating Curve (AUC).

Because attendance was unknown for a considerable number of participants, a sensitivity analysis was performed by repeating Model II with the inclusion of patients with unknown attendance who participated in the end-of-treatment examination in the group with medium/high attendance and people with unknown attendance who did not meet for the end-of-treatment examination in the group with low attendance.

All analyses were performed using STATA MP 15.1 (StataCorp, College Station, Texas, USA).

## Results

A total of 2904 patients were enrolled in the GLA:D® Back programme at 183 clinics from April 2018 to February 2020. The study population for the main analyses consisted of 2395/2904 (82%) responding to the baseline questionnaire, of whom 1690 (71%) also responded to the 3-months follow-up.

### Attendance

**Fig. 1:**
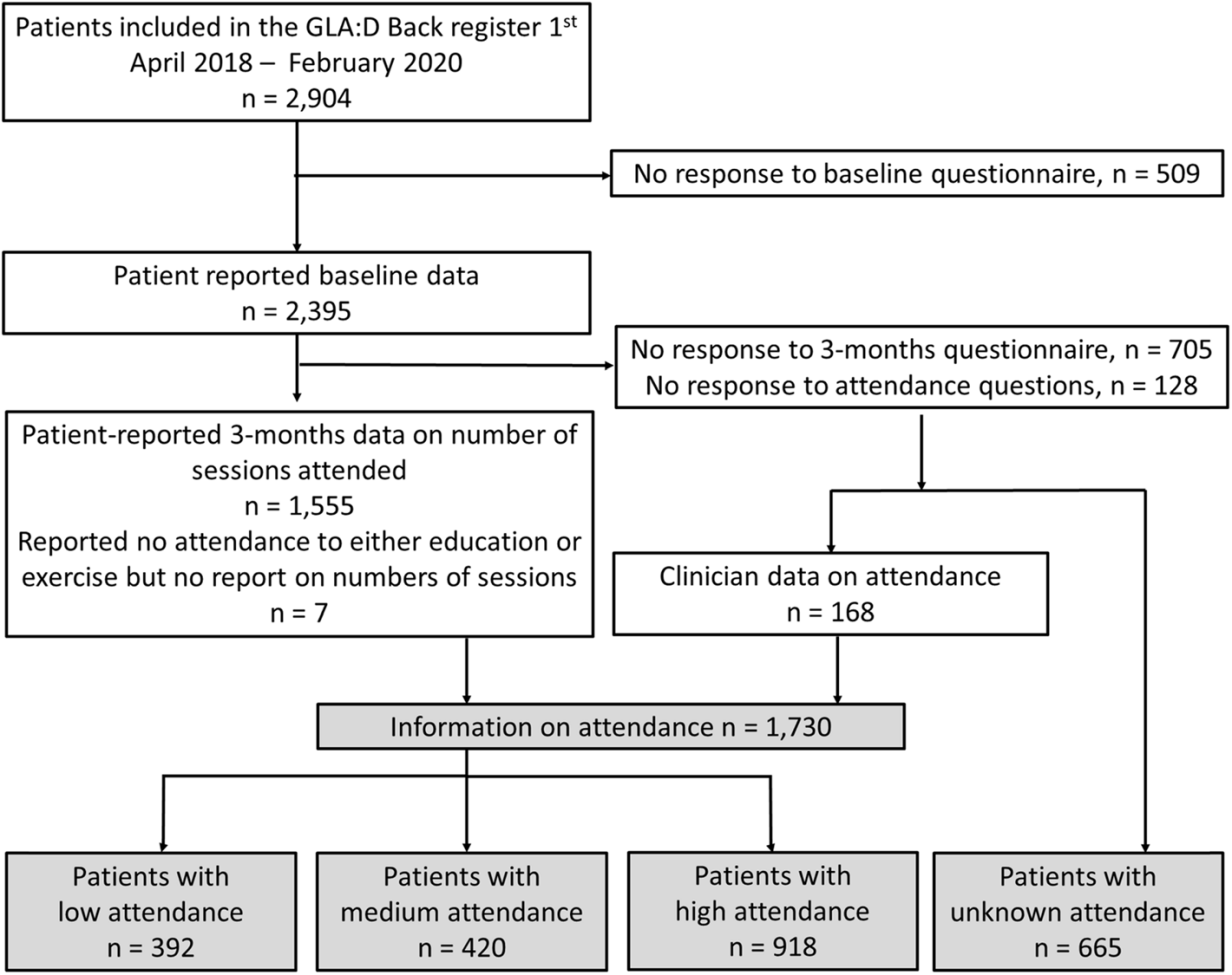
Number of patients in different groups of attendance included in the analyses

Fifty-three per cent (918) had high attendance, 24% (420) medium, and 23% (392) had low attendance (Fig. 1). Most low attending patients did not participate in any education sessions or had only one education session combined with attending between 6 and 10 exercise sessions (Table 1). Most in the medium attendance group only attended one education session (Table 1).

Unknow attendance during the intervention was registered for 28% (665) participants (Fig. 1). From this group, 85 reported participation in GLA:D® Back education *or* exercises sessions (including 39 who participated in both), but not the number of sessions they took part in. Baseline information did not reveal substantial differences between the group with unknown attendance and those with known attendance, although those with unknown attendance were somewhat less likely to be in ordinary work, less likely to be in the STarT low-risk group, and slightly higher back-related disability scored with ODI. A substantially larger proportion of study participants with unknown attendance stopped answering the baseline questionnaire before reaching the end of it compared to those with known attendance (Table 2).

**Table 2:** Characteristics of people within levels of attendance

	High attendance (***n*** = 918)	Medium attendance (***n*** = 420)	Low attendance (***n*** = 392)	Unknown attendance (***n*** = 665)
Sex, % female	69.8	69.1	67.4	70.2
missing %	1.2	0.5	0.5	2.6
Age, median (IQR)	59 (50–67)	59 (49–68)	61 (53–68)	59 (48–68)
missing %	0.4	0.2	1.0	0.9
Education, %				
No qualifying training	39.3	37.9	38.5	40.6
Vocational education	15.3	13.9	13.5	13.6
Higher education (< 5 years)	41.9	43.7	43.5	38.9
Higher education (5+ years)	3.5	4.6	4.4	6.9
missing %	1.5	0.7	2.0	4.1
Work situation, %				
Ordinary work	52.1	47.7	43.0	46.1
Unemployed	2.4	3.4	4.0	3.3
Rehabilitation/Flex job	5.9	3.7	5.8	6.0
Disability pension	3.1	4.1	7.1	5.5
Retired	30.6	31.4	33.0	29.4
Student/trainee/other	5.9	9.7	7.1	9.8
missing %	4.3	2.1	3.3	4.4
Working hours per week^a^, %				
Part-time (< 37 h)	27.9	34.9	32.3	33.6
Full time (37 h)	34.4	30.7	26.0	32.9
> 37 h	37.7	34.4	41.8	33.6
missing %	2.2	2.0	3.1	1.4
Current sick leave^a^, %	5.2	8.6	8.6	7.9
missing %	11.4	11.2	14.1	9.6
Co-morbid pain sites, %				
None	2.7	4.1	2.0	5.7
1–3	59.8	58.0	60.1	52.1
4+	37.8	38.0	38.9	42.2
missing %	9.5	7.1	10.5	17.7
Co-morbid disease, %				
None	15.0	16.4	19.9	14.5
1–2	62.6	58.3	57.0	59.8
3+	22.4	25.3	23.2	25.7
missing %	26.7	25.7	23.0	24.5
Duration of current LBP, %				
< 4 weeks	8.1	10.3	5.4	5.2
4–12 weeks	11.4	11.7	12.8	12.8
3–12 months	22.9	19.6	21.3	22.7
> 12 months	57.7	58.4	60.5	59.4
missing %	1.3	0.5	0.5	3.8
LBP intensity (0–10), median (IQR)	5 (4–7)	6 (4–7)	6 (4–7)	5 (4–7)
missing %	1.0	0.5	0.3	3.0
Leg pain present, %	69.9	76.5	78.8	78.5
missing %	0.8	0.7	0.0	3.5
Start Back Screening Tool, %				
Low risk	51.8	46.9	42.5	39.0
Medium risk	25.8	31.1	26.6	31.7
High risk	22.4	22.0	31.0	29.3
missing %	1.6	0.5	2.0	6.6
Self-efficacy ‘pain’ (0–50), median (IQR)	35 (25–41)	36 (28–41)	34 (27–40)	32 (26–39)
missing %	4.6	2.4	7.1	15.5
Self-efficacy ‘other symptoms’ (0–60), median (IQR)	40 (33–47)	40 (33–48)	39 (30–47)	38 (31–44)
missing %	6.5	3.4	8.2	16.4
Illness Belief (0–80), median (IQR)	43 (35–50)	43 (35–49)	44 (35–52)	45 (37–52)
missing %	2.1	0.7	3.1	8.4
Disability (0–100), median (IQR)	22 (16–32)	24 (16–32)	24 (16–34)	26 (18–34)
missing %	2.0	0.7	2.0	10.2
Extensor endurance (seconds), median (IQR)	78 (40–152)	70 (38–141)	71 (34–144)	65 (31–129)
missing %	0.9	0.7	2.0	2.0
Abdominal endurance (seconds), median (IQR)	47 (25–80)	40 (25–70)	45 (24–74)	43 (23–73)
missing %	1.5	0.7	1.5	2.7
Sit-to-stand test (repetitions), median (IQR)	12 (10–15)	12 (10–14)	12 (10–15)	12 (9–14)
missing %	0.8	0.7	0.8	1.2
Answering of baseline questionnaire stopped before end of survey, %	2.2	0.5	3.1	11.6

Proportions are among those with non-missing data

*LBP* Low back pain, *IQR* Inter Quartile Range

^a^includes only people in ordinary work

### Match between attendance definitions and clinicians’ judgements

The match between definitions of low, medium, high attendance with clinicians’ judgement of attendance was based on information from 342 patients. The results supported the defined criteria for attendance: 89% (167) of participants registered by the clinician as completed the intervention were classified as medium (22%) or high (67%) attendance based upon patient-reported data, while 83% (129) of those who were registered by clinicians to not completed the intervention, were in the low attendance group.

### Patient characteristics within levels of attendance

Differences in patient characteristics between attendance groups were generally small (Table 2). People in a full-time job without sick leave were somewhat more frequent in the high attendance group (52.1% versus 47.7 and 43.0% for high versus medium and low), whereas working > 37 h per week (37.7% versus 34.4 and 41.8% for high versus medium and low) and disability pension (3.1% versus 4.1 and 7.1% for high versus medium and low) were more represented in the low attendance group. For characteristics related to symptoms, very recent onset LBP (< 4 weeks duration) was less frequent among low attenders than in the other groups. Psychological factors such as self-efficacy and Illness Beliefs did not differ between groups (Table 2).

### Factors related to attendance in multivariable models

The multivariable model included 392 participants with low attendance and 1338 with medium/high attendance. Low attendance was most clearly associated with being above 50 years of age, having a STarT high-risk profile, having low levels of self-efficacy for other symptoms than pain, and having less threatening illness perceptions. However, confidence intervals were relatively wide (Table 3). Also, trends were observed for other factors associated with low attendance including shorter duration of symptoms (OR = 1.79 (95% CI 0.97–3.28), and high score on ODI (OR = 1.87 (95% CI 0.47–7.36). The discriminative accuracy based on all the investigated factors was fair (AUC 0.77 (95% CI 0.74–0.79)) (Table 3). Similar discrimination was found based only on the degree to which participants completed the baseline questionnaire (AUC 0.77 (95% CI 0.74–0.79, *n* = 1837). The likelihood low attendance was strongly reduced with responding partially (OR = 0.29 (95% CI 0.12–0.68)) or fully (OR = 0.14 (95% CI 0.9-0.21)) to the questionnaire as compared to not responding at all.

**Table 3:** Multivariable models for factors related to low attendance

	Model I (***n*** = 1730) OR (95% CI)	Model II (***n*** = 1730) OR (95% CI)
Discrimination, AUC (95% CI)	0.77 (0.74–0.79)	0.76 (0.74–0.79)
Sex, % female	0.94 (0.7–1.3)	
Age quartiles		
< 50	reference	0.62 (0.46–0.85)
50–59	1.60 (1.11–2.30)	^a^
60–67	1.51 (1.04–2.18)	
68–88	1.61 (1.07–2.42)	
Education		
No qualifying training	reference	
Vocational education	1.02 (0.69–1.51)	
Higher education (> 3 years)	1.10 (0.83–1.47)	
Other	1.08 (0.57–2.05)	
Work		
Ordinary work, < 37 h	reference	reference
Ordinary work, full time	0.92 (0.65–1.31)	0.92 (0.66–1.30)
Ordinary work, > 37 h	1.19 (0.83–1.69)	1.22 (0.88–1.69)
Rehabilitation/Flex job, < 37 h	0.79 (0.51–1.24)	0.76 (0.49–1.17)
Student/trainee/other	1.46 (0.87–2.46)	1.46 (0.88–2.43)
Co-morbid pain sites		
None	reference	1.24 (0.89–1.72)
1–3	1.67 (0.75–3.70)	^a^
4+	1.45 (0.64–3.30)	
Co-morbid disease		
None	reference	0.61 (0.28–1.34)
1–2	0.82 (0.58–1.15)	^a^
3+	0.74 (0.49–1.11)	
Duration of current LBP		
< 4 weeks	reference	0.67 (0.40–1.12)
4–12 weeks	1.79 (0.97–3.28)	^a^
3–12 months	1.43 (0.81–2.53)	
> 12 months	1.55 (0.91–2.64)	
LBP intensity quartile		
0–4	reference	^a^
4.3–6	1.00 (0.72–1.39)	
6.5–7	0.99 (0.67–1.48)	
7.2–10	1.23 (0.82–1.85)	1.18 (0.85–1.64)
Leg pain present	1.29 (0.95–1.77)	1.26 (0.93–1.70)
Start Back Screening Tool		
Low risk	reference	reference
Medium risk	1.25 (0.88–1.80)	1.18 (0.85–1.65)
High risk	1.73 (1.12–2.69)	1.60 (1.10–2.32)
Self-efficacy pain quartiles		
0–28	reference	1.05 (0.77–1.45)
28–35	0.87 (0.60–1.27)	^a^
35–41	0.89 (0.59–1.33)	
41+	0.97 (0.59–1.60)	
Self-efficacy other symptoms		
0–32	reference	1.48 (1.06–2.08)
32–39	0.57 (0.38–0.83)	^a^
39–47	0.82 (0.54–1.26)	
47+	0.52 (0.31–0.86)	
Illness Belief quartiles		
0–35	reference	1.48 (1.07–2.08)
35–43	0.70 (0.48–1.02)	^a^
43–51	0.63 (0.41–0.97)	
51+	0.56 (0.33–0.93)	
Disability (ODI)		
Minimal	reference	^a^
Moderate	0.97 (0.70–1.34)	
Severe	1.07 (0.64–1.81)	
Crippling	1.87 (0.47–7.36)	1.85 (0.50–6.85)
Extensor endurance (seconds)		
0–36	reference	1.29 (0.94–1.76)
37–72	0.73 (0.50–1.05)	^a^
73–144	0.78 (0.53–1.16)	
145–180	0.87 (0.57–1.32)	
Abdominal endurance (seconds)		
0–25	reference	reference
26–45	0.88 (0.62–1.27)	0.88 (0.62–1.25)
46–75	1.24 (0.85–1.82)	1.28 (0.89–1.86)
76–120	0.81 (0.54–1.23)	0.87 (0.59–1.29)
Sit-to-stand test (repetitions)		
0–10	reference	reference
10–12	0.91 (0.65–1.29)	0.96 (0.68–1.34)
12–14	0.77 (0.53–1.13)	0.78 (0.54–1.13)
14+	1.18 (0.82–1.69)	1.24 (0.87–1.75)

^a^ Other categories of the variable combined into one reference category

**Fig. 2:**
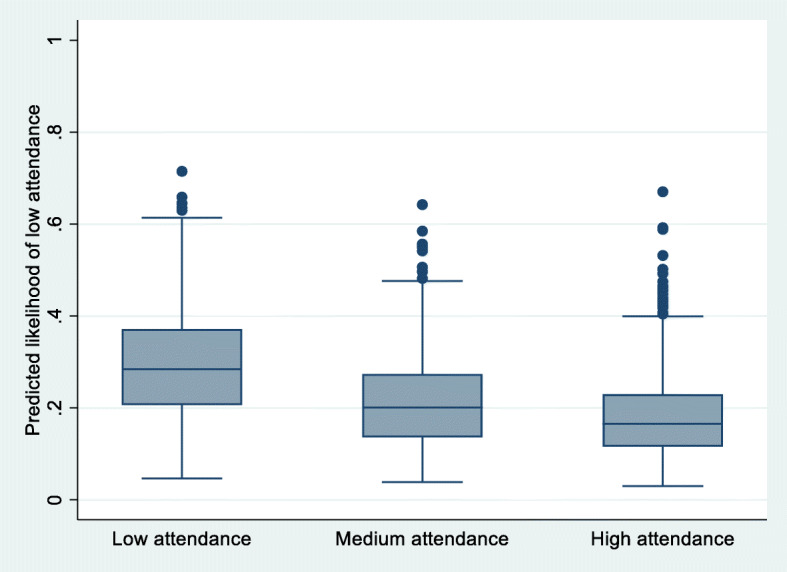
Distributions of predicted likelihood of low attendance in groups of observed attendance in Model II

A considerable overlap between the predicted likelihood of low attendance from Model II across the groups of observed attendance indicated that the model was not useful for predicting attendance in individuals (Fig. 2).

### Sensitivity analysis

The sensitivity analysis including patients with unknown attendance in the group with low attendance if they did not meet for the end-of-treatment examination, and in medium/high attendance if participating in the end-of-treatment examination, did not reveal any other associations between participants characteristics and attendance than observed in the primary analysis, and associations were even weaker (results not presented).

## Discussion

### Summary

Of the participants enrolled in a 10-weeks LBP programme of patient education and exercises, 52% had a high, 23% medium, and 25% low level of attendance. The strongest indicator for the level of attendance was whether participants completed the baseline questionnaire or not, whereas individual baseline patient factors did not reveal any clear pattern. A multivariable model including a range of characteristics and patient-reported outcomes predicted attendance with a moderate accuracy but was not useful to predict at an individual level. Surprisingly, the present study did not show work, level of LBP disability, comorbidities, and response to clinical tests predictive of attendance.

### Other studies

Jordan et al. reported in a Cochrane systematic review, including 42 trials with 8243 patients, that supervised exercises, positive reinforcement, goal setting, problem-solving skills to overcome barriers to adherence and self-monitoring by using an exercise logbook may enhance adherence [29]. These are all integrated elements of the GLA:D® Back programme and therefore not affect adherence in the present study.

Physically related fear avoidance and lower educational level have been reported to reduce adherence in exercise interventions [14, 30]. However, educational level was not related to attendance in the present study perhaps because the content was developed in collaboration with patients, and health literacy was carefully considered.

Meade et al. found that four themes were important for adherence to exercise in persons with persistent musculoskeletal pain: personal, social, and environmental factors as well as the relationship with the physiotherapist [31], and Babatunde scoped the literature and found that therapeutic alliance may influence adherence in musculoskeletal rehabilitation [32]. Finally, using motivational strategies for people with chronic low back pain may improve adherence to the intervention [33]. In the current study, alliance and motivational factors were not studied. Both aspects could be relevant to assess their impact on attendance. The present study does not find any relation between the psychological factors self-efficacy or illness beliefs and attendance. This is noteworthy as self-efficacy cognitions are reported essential determinants of physical activity and exercise behaviour [34–36]. One explanation could be the relatively high out-of-pocket expense for participation in GLA:D Back. Thus, the cohort may be too homogeneous because those unable to afford to participate or who were not highly motivated, were not part of the data collection.

It was not feasible to collect detailed data on how many potential patients eligible to enter the study rejected to participate and for what reasons. Therefore, unknown aspects might influence the composition of this cohort, which can conceal other factors normal applicable to the level of attendance. Also, statistical significance is a function of sample size. Larger sample size would most likely have resulted in more statistically significant results such as the trend towards high-risk scores in the Start Back screening tool being associated with lower attendance, indicating a need for additional psychological interventions for patients with this profile. However, we consider our sample size to be able to detect relevant associations.

Finally, participants used, on average, 26 min to answer the baseline questionnaire. The number of questions and answering time may be a barrier to complete the questionnaire [37].

### Strengths and limitations

The study is practice-based, using patients commonly seen in primary care clinics. Therefore, it has good external validity for people enrolled in a standardised educational/exercise programme for persistent LBP in primary care. The results may be relevant also for other educational/exercise programmes for LPB patients because the results are based upon data from a large number of patients from many primary care clinics across Denmark. Besides, the study informs about patients with unknown attendance, compared with those with different levels of attendance, adding knowledge to this category.

The levels of attendance (high/medium and low) as defined by the research group were clinically validated against clinician reports and corresponded for 89 and 83% for the registration of attendance based upon the criteria for high/medium and low, respectively.

A shortcoming of the study was the patient report of the level of attendance, which could be biased by recall-bias. Real-time registration of attendance was not performed because we prioritised to keep the administrative burden on the clinicians low. Patients have been shown to over-report participation due to desirability and recall bias [38]. Still, we do not consider recall a major source of bias as information about attendance was collected shortly after finishing the programme. The programme’s total duration was 9–10 weeks plus the time between the initial session of testing and goal setting and the beginning of the group-based sessions, meaning that data collection at 3 months was close to its actual ending. Also, patient-reported and clinician-reported data had a high level of agreement, i.e. 89% registered by the clinician as completed were classified as medium or high attendance by the patients, and 83% registered by clinicians not completing the intervention, were in the low attendance group.

The evaluation of attendance was based on 72% (1730) of the patient sample approaching what is considered the minimum acceptable withdrawal rate in clinical trials [39]. As non-response to questionnaires might indicate low adherence to the intervention, we included these patients in the low attendance group in the sensitivity analyses, which did not change our results regarding factors associated with attendance.

Not completing the baseline questionnaire as an indicator for low attendance can imply other factors not measured in this study that influence attendance. People not completing the baseline questionnaire were probably less motivated from the start, which has been shown to be critical in the completion of exercise programs [40]. Also, those with high-risk profiles on the STarT back questionnaire tended to have low attendance. It might be that psychological distress is indicated by a high-risk profile and is associated also with not completing the comprehensive questionnaire. However, we have no data to support or reject that hypothesis.

The success of implementation is complex and multifactorial concerning both clinicians and patients [32, 41–43]. This present study contributes with knowledge on one of these aspects, attendance. Other studies focusing on a variety of patients’ and clinicians’ factors related to implementation of GLA:D Back are in process.

## Conclusions

Most participants of a 10-week LBP education and exercise programme attended almost all sessions. The strongest predictor for low attendance was not completing the baseline questionnaire, which might be an early indicator that can be used to target potential non-compliers. Attendance was not strongly related to patient characteristics. Further qualitative and quantitative research is needed to assess reasons for non-responses of enrolment questionnaires and possibly other factors related to attendance.

## Supplementary Information

**Additional file 1.**

## Data Availability

The datasets used and/or analysed during the current study are available from the corresponding author on reasonable request.

## Abbreviations

LBP: Low back pain
ASES: Arthritis Self-efficacy Scale
B-IPQ: Brief Illness Perception Questionnaire
ODI: Oswestry Disability Index
IQR: Interquartile Ranges
OR: Odds Ratios
CI: Confidence Intervals
AUC: Area Under the Receiver Operating Curve
