# Supplementary material for: Adherence and characteristics of participants enrolled in a standardised programme of patient education and exercises for low back pain, GLA:D® Back – a prospective observational study
Source: BMC Musculoskelet Disord. 2021 May 22;22:473. doi: 10.1186/s12891-021-04329-y (PMC8141215; doi:10.1186/s12891-021-04329-y)
Supplement: Supplementary file 1 — Additional file 1. [file 12891_2021_4329_MOESM1_ESM.docx]

**Questionnaires used in *Adherence and characteristics of participants enrolled in a standardised programme of patient education and exercises for low back pain, GLA:D® Back – a prospective observational study***

Oswestry Disability Index

| oswestry1 | Section 1: Pain | 1, I have no pain at the moment \| 2, The pain is very mild at the moment \| 3, The pain is moderate at the moment \| 4, The pain is fairly severe at the moment \| 5, The pain is very severe at the moment \| 6, The pain is the worst imaginable at the moment |
| --- | --- | --- |
| oswestry2 | Section 2: Personal care (eg washing, dressing etc) | 1, I can look after myself normally without causing extra pain \| 2, I can look after myself normally, but it causes extra pain \| 3, It is painful to look after myself and I am slow and careful \| 4, I need some help but manage most of my personal care \| 5, I need help every day in most aspects of self-care \| 6, I do not get dressed, I wash with difficulty and stay in bed |
| oswestry3 | Section 3: Lifting | 1, I can lift heavy weights without extra pain \| 2, I can lift heavy weights but it gives extra pain \| 3, Pain prevents me from lifting heavy weights off the floor, but I can manage if they are conveniently placed eg. on a table \| 4, Pain prevents me from lifting heavy weights, but I can manage light to medium weights if they are conveniently positioned \| 5, I can lift very light weights \| 6, I cannot lift or carry anything at all |
| oswestry4 | Section 4: Walking | 1, Pain does not prevent me walking any distance \| 2, Pain prevents me from walking more than 1 mile \| 3, Pain prevents me from walking more than 1/2 mile \| 4, Pain prevents me from walking more than 100 yards \| 5, I can only walk using a stick or crutches \| 6, I am in bed most of the time |
| oswestry5 | Section 5: Sitting | 1, I can sit in any chair as long as I like \| 2, I can only sit in my favourite chair as long as I like \| 3, Pain prevents me sitting more than one hour \| 4, Pain prevents me from sitting more than 30 minutes \| 5, Pain prevents me from sitting more than 10 minutes \| 6, Pain prevents me from sitting at all |
| oswestry6 | Section 6: Standing | 1, I can stand as long as I want without extra pain \| 2, I can stand as long as I want but it gives me extra pain \| 3, Pain prevents me from standing for more than 1 hour \| 4, Pain prevents me from standing for more than 30 minutes \| 5, Pain prevents me from standing for more than 10 minutes \| 6, Pain prevents me from standing at all |
| oswestry7 | Section 7: Sleeping | 1, My sleep is never disturbed by pain \| 2, My sleep is occasionally disturbed by pain \| 3, Because of pain I have less than 6 hours sleep \| 4, Because of pain I have less than 4 hours sleep \| 5, Because of pain I have less than 2 hours sleep \| 6, Pain prevents me from sleeping at all |
| oswestry8 | Section 8: Sex Life (if applicable) | 1, My sex life is normal and causes no extra pain \| 2, My sex life is normal but causes some extra pain \| 3, My sex life is nearly normal but is very painful \| 4, My sex life is severely restricted by pain \| 5, My sex life is nearly absent because of pain \| 6, Pain prevents any sex life at all |
| oswestry9 | Section 9: Social life | 1, My social life is normal and gives me no extra pain \| 2, My social life is normal but increases the degree of pain \| 3, Pain has no significant effect on my social life apart from limiting my more energetic interests eg, sport \| 4, Pain has restricted my social life and I do not go out as often \| 5, Pain has restricted my social life to my home \| 6, I have no social life because of pain |
| oswestry10 | Section 10: Travelling | 1, I can travel anywhere without pain \| 2, I can travel anywhere but it gives me extra pain \| 3, Pain is bad but I manage journeys over two hours \| 4, Pain restricts me to journeys of less than one hour \| 5, Pain restricts me to short necessary journeys under 30 minutes \| 6, Pain prevents me from travelling except to receive treatment |

Start Back Screening Tool

| start1 | Has your back pain spread down your leg(s) at some time in the last 2 weeks? | 0, No \| 1, Yes |
| --- | --- | --- |
| start2 | Have you had pain in the shoulder or neck at some time in the last 2 weeks | 0, No \| 1, Yes |
| start3 | Have you only walked short distances because of your back pain? | 0, No \| 1, Yes |
| start4 | In the last 2 weeks, have you dressed more slowly than usual because of back pain? | 0, No \| 1, Yes |
| start5 | Do you think it's not really safe for a person with a condition like yours to be physically active? | 0, No \| 1, Yes |
| start6 | Have worrying thoughts been going through your mind a lot of the time? | 0, No \| 1, Yes |
| start7 | Do you feel that your back pain is terrible and it's never going to get any better? | 0, No \| 1, Yes |
| start8 | In general, have you stopped enjoying all the things you usually enjoy? | 0, No \| 1, Yes |
| start9 | Overall, how bothersome has your back pain been in the last 2 weeks? | 1, Not at all \| 2, Slightly \| 3, Moderately \| 4, Very much \| 5, Extremely |

Arthritis Self-efficacy Scale

| ases1 | How certain are you that you can decrease your pain quite a bit? | Very uncertain 0 \| 1 \| 2 \| 3 \| 4 \| 5 medium certain \| 6 \| 7 \| 8 \| 9 \| 10, Very certain |
| --- | --- | --- |
| ases2 | How certain are you that you can continue most of your daily activities? | Very uncertain 0 \| 1 \| 2 \| 3 \| 4 \| 5 medium certain \| 6 \| 7 \| 8 \| 9 \| 10, Very certain |
| ases3 | How certain are you that you can keep your pain from interfering with your sleep? | Very uncertain 0 \| 1 \| 2 \| 3 \| 4 \| 5 medium certain \| 6 \| 7 \| 8 \| 9 \| 10, Very certain |
| ases4 | How certain are you that you can that you can make a small-to-moderate reduction in your back pain by using methods other than taking extra medication? | Very uncertain 0 \| 1 \| 2 \| 3 \| 4 \| 5 medium certain \| 6 \| 7 \| 8 \| 9 \| 10, Very certain |
| ases5 | How certain are you that you can make a large reduction in your low back pain by using methods other than taking extra medication? | Very uncertain 0 \| 1 \| 2 \| 3 \| 4 \| 5 medium certain \| 6 \| 7 \| 8 \| 9 \| 10, Very certain |
| ases6 | How certain are you that you can control your fatigue? | Very uncertain 0 \| 1 \| 2 \| 3 \| 4 \| 5 medium certain \| 6 \| 7 \| 8 \| 9 \| 10, Very certain |
| ases7 | How certain are you that you can regulate your activity so you can be active without aggravating the your back problem? | Very uncertain 0 \| 1 \| 2 \| 3 \| 4 \| 5 medium certain \| 6 \| 7 \| 8 \| 9 \| 10, Very certain |
| ases8 | How certain are you that you can do something to help yourself feel better if you are feeling blue? | Very uncertain 0 \| 1 \| 2 \| 3 \| 4 \| 5 medium certain \| 6 \| 7 \| 8 \| 9 \| 10, Very certain |
| ases9 | As compared with other people with back problems like yours, how certain are you that you can manage your pain during your daily activities? | Very uncertain 0 \| 1 \| 2 \| 3 \| 4 \| 5 medium certain \| 6 \| 7 \| 8 \| 9 \| 10, Very certain |
| ases10 | How certain are you that you can manage your back problem so that you can do the things you enjoy doing? | Very uncertain 0 \| 1 \| 2 \| 3 \| 4 \| 5 medium certain \| 6 \| 7 \| 8 \| 9 \| 10, Very certain |
| ases11 | How certain are you that you can deal with the frustration of back problems? | Very uncertain 0 \| 1 \| 2 \| 3 \| 4 \| 5 medium certain \| 6 \| 7 \| 8 \| 9 \| 10, Very certain |

Brief Illness Perception Questionnaire

| bipq1 | How much does your back problem affect your life? | No affect at all 0 \| 1 \| 2 \| 3 \| 4 \| 5 \| 6 \| 7 \| 8 \| 9 \| 10 Severly affects my life |
| --- | --- | --- |
| bipq2 | How long do you think your back problem will continue? | No affect at all 0 \| 1 \| 2 \| 3 \| 4 \| 5 \| 6 \| 7 \| 8 \| 9 \| 10 Severly affects my life |
| bipq3 | How much control do you feel you have over your back problem? | No affect at all 0 \| 1 \| 2 \| 3 \| 4 \| 5 \| 6 \| 7 \| 8 \| 9 \| 10 Severly affects my life |
| bipq4 | How much do you think your treatment can help your back problem? | No affect at all 0 \| 1 \| 2 \| 3 \| 4 \| 5 \| 6 \| 7 \| 8 \| 9 \| 10 Severly affects my life |
| bipq5 | How much do you experience symptoms from your back problems? | No affect at all 0 \| 1 \| 2 \| 3 \| 4 \| 5 \| 6 \| 7 \| 8 \| 9 \| 10 Severly affects my life |
| bipq6 | How concerned are you about your back problem? | No affect at all 0 \| 1 \| 2 \| 3 \| 4 \| 5 \| 6 \| 7 \| 8 \| 9 \| 10 Severly affects my life |
| bipq7 | How well do you feel you understand your back problem? | No affect at all 0 \| 1 \| 2 \| 3 \| 4 \| 5 \| 6 \| 7 \| 8 \| 9 \| 10 Severly affects my life |
| bipq8 | How much does your back problem affect you emotionally? (e.g. does it make you angry, scared, upset or depressed? | No affect at all 0 \| 1 \| 2 \| 3 \| 4 \| 5 \| 6 \| 7 \| 8 \| 9 \| 10 Severly affects my life |
| bipq_cause1 | 1. (most important factors causing back problem) | No affect at all 0 \| 1 \| 2 \| 3 \| 4 \| 5 \| 6 \| 7 \| 8 \| 9 \| 10 Severly affects my life |
| bipq_cause2_ | 2. (most important factors causing back problem) | No affect at all 0 \| 1 \| 2 \| 3 \| 4 \| 5 \| 6 \| 7 \| 8 \| 9 \| 10 Severly affects my life |
| bipq_cause3 | 3. (most important factors causing back problem) | No affect at all 0 \| 1 \| 2 \| 3 \| 4 \| 5 \| 6 \| 7 \| 8 \| 9 \| 10 Severly affects my life |
